# Treatment effect measures for culture conversion endpoints in phase IIb tuberculosis treatment trials

**DOI:** 10.1093/cid/ciab576

**Published:** 2021-12-06

**Authors:** Isabelle R. Weir, Sean Wasserman

**Affiliations:** 1 Center for Biostatistics in AIDS Research in the Department of Biostatistics, Harvard T.H. Chan School of Public Health, Boston, MA; 2 Wellcome Centre for Infectious Diseases Research in Africa, Institute of Infectious Disease and Molecular Medicine, University of Cape Town, Cape Town, South Africa; 3 Division of Infectious Diseases and HIV Medicine, Department of Medicine, University of Cape Town, Cape Town, South Africa

**Keywords:** Randomized controlled trials, Tuberculosis, Clinical Trials, Phase II, Treatment Outcome, Survival analysis

## Abstract

Phase IIb trials of tuberculosis therapy rely on early biomarkers of treatment effect. Despite limited predictive ability for clinical outcomes, culture conversion, the event in which an individual previously culture positive for *Mycobacterium tuberculosis* yields a negative culture after initiating treatment, is a commonly used endpoint. Lack of consensus on how to define the outcome and corresponding measure of treatment effect complicates interpretation and limits between-trial comparisons. We review common analytic approaches to measuring treatment effect and introduce difference in restricted mean survival times as an alternative to identify faster times to culture conversion and express magnitude of effect on the time scale. Findings from the PanACEA MAMSTB trial are reanalyzed as an illustrative example. In a systematic review we demonstrate variability in analytic approaches, sampling strategies, and outcome definitions in phase IIb tuberculosis trials. Harmonization would allow for larger meta-analyses, and may help expedite advancement of new TB therapeutics.

## Introduction

Tuberculosis (TB) remains a critical public health concern and leading cause of death worldwide. Although effective therapy exists, regimens for both drug-sensitive (DS) and resistant (DR)-TB require an intensive commitment of at least six-months [1]. Over the last decade, randomized trials for TB treatment have evaluated novel, shorter regimens. A successful shorter regimen should demonstrate acceptable efficacy with comparable cure rates and prevention of long-term relapse, measured at least six-months after the end of treatment. Definitive phase III trials take several years to complete and require large sample sizes to confirm non-inferiority of experimental regimens on clinical endpoints. Smaller phase II trials help identify experimental regimens with a reasonable prospect of clinical success, justifying the enormous investment needed for phase III evaluation. Absent a validated surrogate endpoint for clinical TB outcomes, these trials rely on putative intermediate endpoints which are early biomarkers of treatment response, and assume the difference between interventions on these endpoints are likely to reflect differences on an important clinical endpoint, such as treatment failure or long-term relapse [2].

The most commonly used intermediate endpoint is culture conversion, which is achieved if an individual culture positive for *Mycobacterium tuberculosis* yields a negative culture after initiating treatment. There are empirical associations between culture conversion and treatment success, failure, and recurrence in TB [3]. The proportion remaining culture positive at two months consistently correlates with relapse rates and has been advocated as an intermediate endpoint for DS-TB trials [3–5], but there are major limitations to this approach. A single measurement dichotomous outcome does not capture the biology of mycobacterial killing over time [6] and is a poor discriminant of treatment outcome within and across trial populations [2, 7]. Another drawback is lower statistical power compared with a continuous outcome. Therefore most TB treatment trials are now designed using time to culture conversion [7] which provides information across the biphasic treatment response [6]. However, time to culture conversion lacks adequate precision to be considered a true surrogate of treatment success [8]; earlier time to culture conversion failed to predict successful 4-month fluoroquinolone regimens [9] and had limited use as a surrogate endpoint in the REMoxTB trial [7].

There are complexities to consider with culture conversion endpoints. First, there is no consensus on a time horizon for culture conversion achievement. Trials specify a time horizon logistically feasible and reasonable regarding the experimental drug regimen. Second, time-to-event outcomes require longitudinal assessments but there is no harmonization for the frequency of these culture collections, which may be prone to missing data or misidentification of when culture conversion is achieved. Third, trials must define 'stable' culture conversion, as a single negative culture may not be reliable due to the inherent risk of specimen contamination. Finally, trials are designed to either assess whether culture conversion has occurred at a prespecified time (eg, 2-month culture conversion) or measure the time until culture conversion occurs. Within these two frameworks, there are several treatment effect measures to assess efficacy and promise for a subsequent phase III trial. Misinterpretation of findings based on measures of effect may be common and lead to misinformed conclusions [10]. Despite these limitations, and absent other validated pathogen- or host-based surrogate markers, phase IIb trials continue to use culture conversion primary endpoints as the best available marker of treatment response and it is essential that appropriate analytical approaches are applied and correctly interpreted.

This paper aims to review common measures of treatment effect to aid design and interpretation of phase IIb TB trials using culture conversion endpoints as a primary outcome. We introduce difference in restricted mean survival times (RMST) as an alternative effect measure and reanalyze findings from the PanACEA MAMS-TB trial as an illustrative example. We also perform a systematic search of phase IIb TB trials to describe treatment effect measures, sampling strategies, and outcome definitions to identify areas for harmonization.

## Treatment Effect Measures For Comparing Treatment Arms

### Contrast of culture conversion probabilities at time τ

Basic measures of treatment effect, including difference in probabilities or odds ratio, compare proportions of participants achieving culture conversion at time *τ* based on a single culture collection. Larger trials focused on differences in proportions may use Chi-squared or likelihood-ratio tests while smaller trials use Fisher’s exact test. Trials analyzing an odds ratio use a Mantel-Haenszel test or logistic regression. Analysis populations and plans addressing potential non-random loss to follow-up should be pre-specified. For example, lost participants may have been less likely to experience culture conversion, leading to falsely inflated proportions if excluded from the analysis.

Proportions offer a simple interpretation and may be used to screen out poorly performing regimens in Phase IIb trials, but have drawbacks. They quantify the treatment effect at a single instance providing no information about timing of effects. Potentially important treatment response measures which may translate into longer term efficacy, such as a shorter time to culture conversion, are therefore missed. Culture conversion rates are high with the standard regimen, and relatively large sample sizes are required to show a meaningful difference with an experimental regimen. A comparison of proportions has inherently less statistical power (and therefore requires larger samples) than tests using time-to-event data.

### Difference in cumulative probability of culture conversion by time τ

With time-to-event data cultures must be collected longitudinally to identify the time when conversion occurred. Trials using this approach may be designed using a Cox’s proportional hazards model or log-rank test. The test using Cox’s proportional hazards model assesses whether the coefficient for treatment differs from the null value of 0 (i.e. no effect) at time τ. This model relies on assumptions including proportional hazards (i.e. hazard ratio remains constant over time) and non-informative (independent) censoring. Log-rank tests use all follow-up data to assess a difference in the distribution of times to culture conversion between two arms. This test also assumes non-informative censoring. It is most powerful when the proportional hazards assumption is met.

The cumulative probability of culture conversion at time τ can be estimated from trial data with the Kaplan-Meier estimator. The cumulative probability gives the probability of culture conversion at time τ conditioned on the fact that culture conversion has not yet occurred. These estimates should be reported for each arm, with differences accompanied by a corresponding confidence interval. A figure showing Kaplan-Meier curves displays time-to-event distributions, allowing the reader to see how the cumulative probabilities change over time, and providing useful insights about differential timing of culture conversion by arm. However, the point estimate of the difference in cumulative probabilities quantifies the treatment effect at a single time point and does not capture differences that may have varied prior to time τ.

### Hazard ratio (HR)

HR measures the instantaneous probability of culture conversion in the experimental arm relative to the control arm. Estimation relies upon an underlying assumption that hazard functions for the two arms remain proportional over time. This assumption is assessed by visual assessment of plotted Schoenfeld residuals or a Grambsch-Therneau test.

HR is estimated by the exponentiated coefficient for the treatment variable in a Cox’s proportional hazards model. We can say that on a given day, an individual still presenting as culture positive on the experimental treatment is *HR* times as likely to experience culture conversion compared with a control arm individual. The HR is frequently misinterpreted; it is important to clarify that HR quantifies the *relative treatment effect at a single time point* [10]. An HR greater than 1 implies that more participants on the experimental regimen achieved culture conversion at time τ, implying faster culture conversion, but it does not quantify the reduction in time to conversion. Absolute measures of effect (such as differences) offer more intuitive interpretation and may be more beneficial for public health decision making [11, 12].

If the proportional hazards assumption does not hold, then the HR estimate should be interpreted with caution as it is really an average effect through time, smoothing over time-varying HRs from different time intervals with potentially misleading conclusions [11]. The proportional hazards assumption is not assured to hold in TB treatment trials. Over a treatment duration, experimental therapies may yield differential hazard rates of culture conversion compared with standard therapy for certain time intervals. For example, optimized rifampicin or experimental agents such as delamanid and quinolones have a dose-response relationship for early bactericidal activity (EBA) resulting in more rapid early killing; other experimental agents like clofazimine mediate activity through sterilising effect with delayed killing [13, 14].

### Difference in median times to culture conversion

Median time to culture conversion provides an estimate of the time at which half of participants on a treatment arm have experienced the outcome. TB trial design with medians as a primary outcome is uncommon, and median times are often supplemental. Median times are estimated from a Kaplan-Meier estimator; the Wilcoxon Rank Sum test can be used to make a formal comparison. Confidence intervals for the difference in median times involve complex methods and often lack precision [15].

Estimation of the median times requires at least half of randomized participants on each respective arm experience culture conversion by time τ. Standard of care treatment has a high efficacy rate on culture conversion for DS-TB, so this requirement is typically expected to be met after 4-6 weeks of therapy. The median difference captures treatment effect at a single instance and is not sensitive to early or late differences between two treatment arms. Comparisons of medians between trials have limited utility because median culture conversion times are strongly influenced by study visit schedules.

### Difference in restricted mean times to culture conversion (RMST)

Difference in RMST is a less familiar measure of treatment effect for time-to-event outcomes [16]. The RMST captures the expected time to culture conversion for a participant followed up to a pre-specified, clinically relevant, time horizon, τ, coinciding with the end of experimental TB treatment or the selected surrogate outcome (eg, 2-month culture conversion). RMST is given by the area under the survival function, often estimated by the Kaplan-Meier estimator [Appendix Text 1]. The difference in RMST over time 0 to τ gives the expected reduction in time to culture conversion for those on experimental compared with control regimen. Unlike differences in cumulative probabilities which give an estimate at a single time point, it summarizes the treatment effect over a time interval. There are many shapes of Kaplan-Meier curves with the same estimated difference in cumulative probability at time τ that potentially give different estimates of corresponding differences in RMST. A test of means assesses whether the difference in RMST differs from the null effect of 0. Prior work demonstrated agreement between tests for the difference in RMST and the log-rank test [17–19]. RMST can be adjusted for covariates using methods such as inverse probability weighting or regression based approaches [20–22].

The difference in RMST does not rely on underlying model assumptions, such as proportional hazards. It reveals faster (or slower) times to culture conversion for an experimental regimen over a full time interval rather than at a single instance and quantifies the magnitude of effect in easily understood time units, such as days (Table 1). Biostatisticians have recently promoted this treatment effect measure for oncology and cardiovascular trials [17, 23]. Besides one reanalysis of time to virologic failure in an HIV treatment trial [24] the difference in RMST has not, to our knowledge, been proposed for the analysis of time-to-event outcomes in infectious disease trials.

### Illustrative example of RMST: PanACEA MAMS TB trial

To illustrate the application of RMST, we selected a phase IIb trial from a literature search that included a high quality graphic of Kaplan-Meier curves for times to culture conversion [25]. We used DigitizeIt software (http://www.digitizeit.de/) to reconstruct individual level data from each arm using the time and probability coordinates from the published Kaplan-Meier curves [Appendix Text 2] [26].

The PanACEA multi-arm, multi-stage (MAMS) trial evaluated whether rifampicin at higher doses or in combination with new/repurposed drugs was associated with treatment-shortening potential in DS-TB [25]. The open-label trial randomized 365 participants with pulmonary TB to receive standard DS-TB therapy or one of four experimental regimens. The primary endpoint was time to liquid culture conversion at 12 weeks, analysed using an adjusted Cox proportional-hazards model. The trial was powered to detect a HR of 1.8, the assumed effect size indicative of treatment-shortening potential. Investigators reported numerically higher cumulative probability of culture conversion at 12 weeks (79.9% vs. 70.1%) and shorter median time to culture conversion (48 vs. 62 days), with an adjusted HR of 1.78 (95% confidence interval, 1.22 -2.58) in favor of the highest dose rifampicin arm (R35HZE) compared with control. This led to a conclusion that “a regimen including rifampicin 35 mg/kg resulted in significantly faster liquid culture conversion by 12 weeks.”

**Figure 1:**
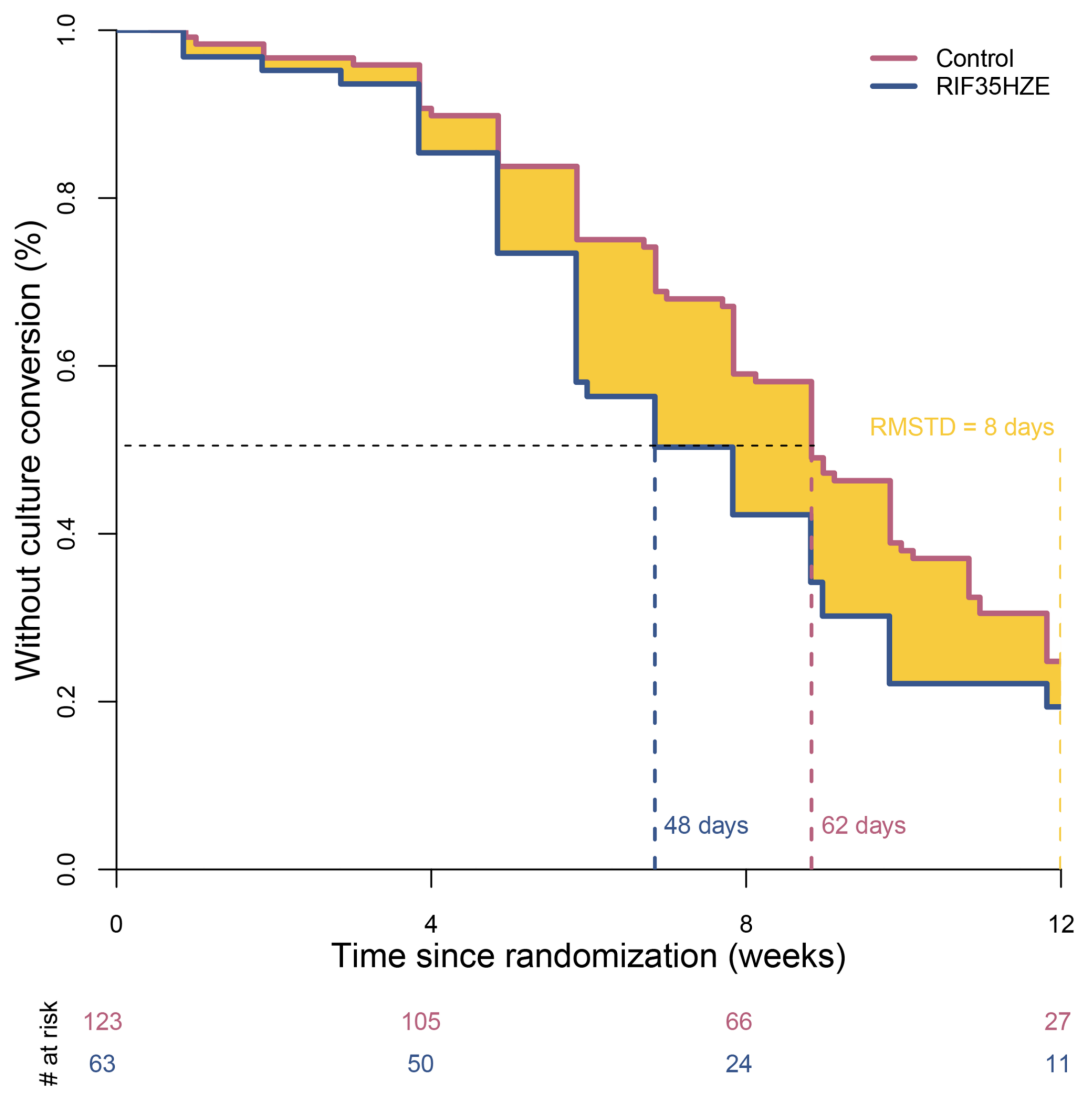
Time to culture conversion, PanACEA MAMS TB trial Time to stable culture conversion in liquid media, up to 12 weeks, in the modified intent to treat (mITT) population based on reconstructed data. The cumulative probability of culture conversion by 12 weeks was 0.80 (95% CI 0.66 to 0.89) in the R35HZE arm and 0.70 (95% CI 0.60 to 0.77) in the control arm [Appendix Text 2]. The median time to culture conversion is 48 days in the R35HZE arm and 62 days in the control arm. The unadjusted hazard ratio is 1.50 (95% CI 1.06 to 2.20) meaning that on a given day, an individual still presenting as culture positive on the R35HZE regimen is 1.50 times as likely to experience culture conversion compared with an individual on the control regimen. The difference in RMST over 12 weeks of follow-up is 8 days (95% CI 1 to 15 days) meaning that over 12 weeks of follow-up, those on the R35HZE regimen experienced culture conversion 8 days faster on average as compared with those on the standard regimen. Dashed lines indicate the median culture conversion times in each arm and the time horizon for the difference in RMST (12 weeks). The shaded area between the Kaplan-Meier curves represents the estimated difference in RMST, 8 days.

As discussed, HR implies faster culture conversion but does not quantify reduction in time. Median times capture an effect at a single instance based only on 50% of participants achieving conversion in each arm. Trials often report faster time to culture conversion based on these measures of effect with potential for misinterpretation. In contrast, the difference in RMST merits this desired conclusion and provides an estimate of the magnitude of effect on the time scale using all data up to time τ. We reconstructed the MAMS trial data for time to culture conversion comparing the R35HZE arm with control, finding agreement with reported HR and median days to culture conversion (Figure 1). For the time horizon of 12 weeks, the RMSTs were 23 and 31 days for the R35HZE and control arms, respectively. The difference in RMST was 8 days (95% CI 1 to 15 days), substantiating a conclusion that over 12 weeks of treatment, those in the R35HZE arm experienced faster times to culture conversion (by 8 days on average) compared with those in the control arm (p-value=0.02).

In our reanalysis, the sizeable HR estimate in the MAMS trial (1.46) translated to only a small reduction in time to culture conversion (8 days) using all data through 12 weeks. A major knowledge gap in TB regimen evaluation is the effect size for culture conversion endpoints that translates into clinical outcomes. There is reliance on seemingly large relative effect sizes in phase IIb trials with the underlying assumption that this reflects treatment effect on long-term efficacy. While an 8-day reduction in mean time to culture conversion over 12 weeks may ultimately predict treatment shortening with high dose rifampicin, this finding tempers enthusiasm based on a large relative HR effect.

## Review of Phase IIb TB Trials

**Figure 2:**
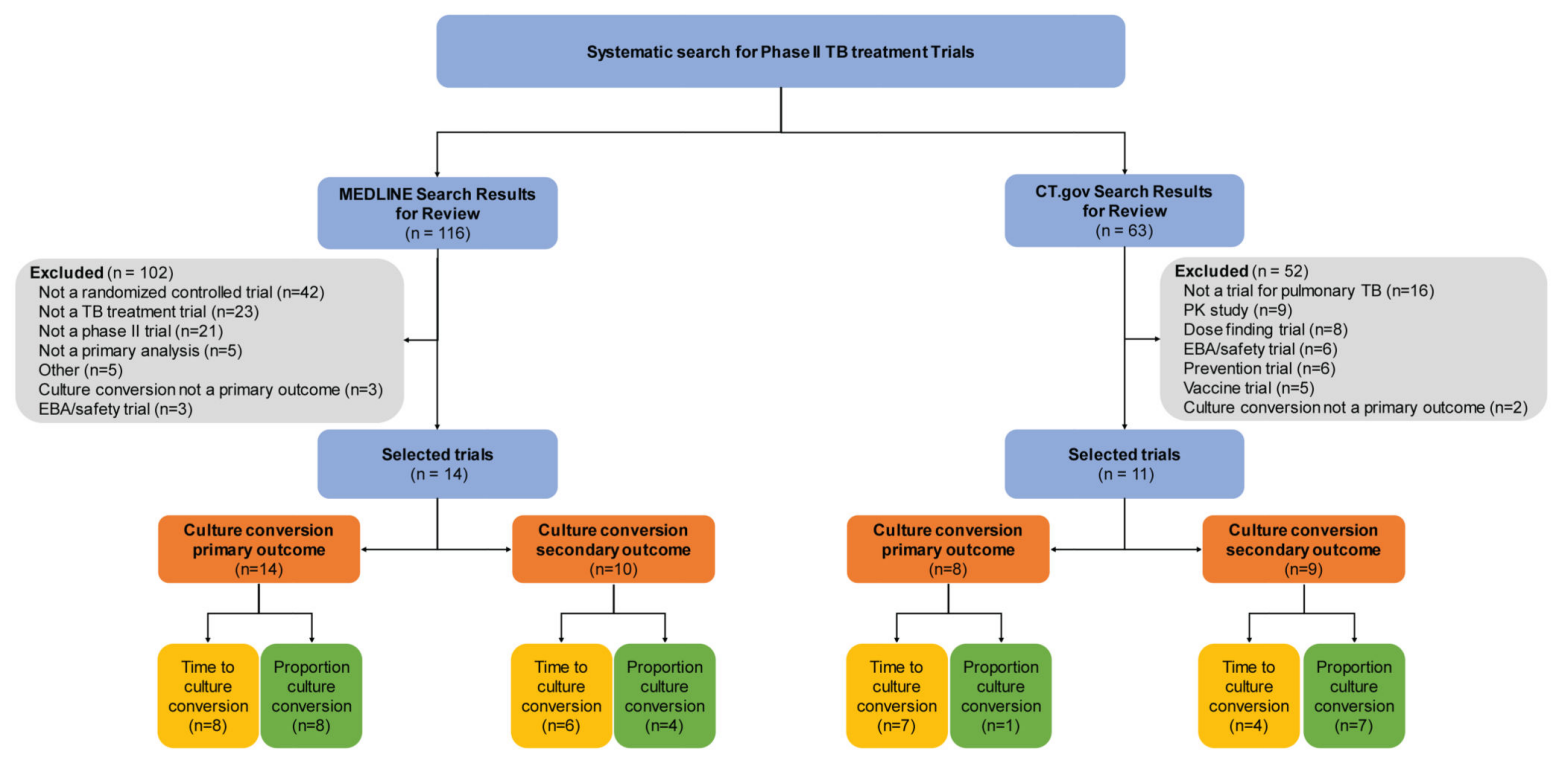
Trial Selection Flow

To evaluate design and analytical practices, we systematically searched MedLine and ClinicalTrials.gov identifying phase IIb randomized controlled trials for pulmonary TB treatment [details in Appendix Text 3]. The Pub Med [Appendix Text 4] and ClinicalTrials.gov searches yielded 116 published manuscripts and 63 trial registrations for consideration. Figure 2 shows the selection process leading to a sample of 25 trials (14 published, 11 registered). The mean sample size was 238 (standard deviation, 163).

Fifteen (60%) trials reported a definition of stable culture conversion; most required more than one negative culture to confirm conversion, using the first negative culture as the time of culture conversion (Tables 2 and 3). Twelve (86%) published trials reported culture collection time points (visits). Many trials collected weekly cultures through six weeks of treatment before reducing to every other week or longer; several collected sputum for culture at wider 4-week, or even 12-week, intervals. The most common time horizon for primary culture conversion outcomes was 8 weeks (11/25 trials, range 4-104 weeks).

Among published trials, 8 (57%) reported time to culture conversion and 8 (57%) reported the proportion achieving culture conversion by a specified time as the primary or co-primary outcome (Table 2). Seven (64%) registered trials use time to culture conversion as a primary outcome while only one trial (9%) uses a proportion (Table 3). For time to culture conversion outcomes among the 25 trials, 9 (36%) report median times (1 trial formally tests differences) and 6 (24%) trials report HR (only 1 (4%) trial reported proportional hazards assessment (using Schoenfeld residuals)). Eight (32%) trials use the log-rank test to compare the distribution of time to culture conversion between two arms. For dichotomous culture conversion outcomes, most trials used Fisher’s exact (or Chi square) test and report simple proportions. Two trials report odds ratios estimated from logistic regression models. Secondary outcomes included both dichotomous and time-to-event endpoints; many trials have effect measures addressing both outcome definitions (Appendix Tables 1 and 2).

## Conclusions

Culture conversion endpoints are limited surrogates for TB relapse. New methodological approaches have been proposed to identify other potential phase IIb trial endpoints. One group used mathematical modeling to derive microbial kill slopes for persister mycobacterial populations (the target of sterilizing activity) from time to culture positivity data, and derived thresholds highly predictive of relapse-free cure [27]. This requires external validation. For now, culture conversion remains the standard for phase IIb TB trials, and it is crucial that associated analytical approaches are correctly applied and interpreted. We found variability in culture conversion definitions, collection times, time horizons, and reported measures of treatment effect. Some variability is expected and may be driven by trial-specific factors, but harmonization would allow for between-trial comparisons and larger meta-analyses, and may help expedite advancement of new TB therapeutics. More work is needed to understand how magnitude of treatment effects using culture-based endpoints translate into clinical outcomes, including definition of effect size targets and optimal sampling strategies. Previous surrogacy validations of culture conversion have focused on the dichotomous, not time-to-event, outcome. Difference in RMST is an absolute measure without underlying model assumptions capturing the treatment effect over a full time interval rather than at a single instance. The unique interpretation quantifies faster times to culture conversion in familiar time units. It is plausible that large differences in RMST for time to culture conversion may be a better predictor of long-term clinical outcomes than relative measures like HR. Future phase IIb TB treatment trials using a culture conversion endpoint should consider supplementing analysis reports with this attractive measure of effect.

## Supplementary Material

Supplementary Material

## Figures and Tables

**Table 1:** Advantages and disadvantages of different measures of treatment effect

Measure	Interpretation of MAMS Trial findings	Advantages	Disadvantages
**Contrast of culture conversion probabilities**	There is a 1% difference in the rate of culture conversion by 26 weeks between the two regimens with 51(81%) participants on the experimental regimen and 101(82%) participants on the comparator regimen experiencing culture conversion.	Simple to calculate and understand Cultures can be collected only at the time point of interest	Captures findings at a single instance in time (not informative about potential for subsequent culture conversion) Requires definition of analysis population for handling participants lost to follow-up
**Difference in cumulative probability of culture conversion**	Participants on the experimental regimen have a 5% higher absolute difference in cumulative probability of experiencing culture conversion by 12 weeks compared to those on the control regimen.	Easily read off survival curves Accounts for censoring	Requires repeated culture collections over time
**Hazard ratio (HR)**	On a given day, an individual still presenting as culture positive on the experimental regimen is 1.5 times as likely to experience culture conversion compared with an individual on the control regimen.	Clinicians are familiar with trial design for a hazard ratio	Difficult to interpret Does not provide an interpretation on the time scale or as a probability of culture conversion in each arm Requires hazard functions to be proportional over time Relative (rather than absolute) measure of effect Requires repeated culture collections over time
**Difference in median times to culture conversion**	The median time to culture conversion was 14 days earlier for those on the experimental regimen as compared with those on the control regimen.	Quantifies treatment effect on time scale Easily read off survival curves	Requires at least 50% of participants in each treatment arm experience culture conversion in the follow-up period Requires repeated culture collections over time Influenced by visit schedule Captures findings at a single instance in time (not informative about potential for subsequent culture conversion)
**Difference in restricted mean times to culture conversion (RMST)**	Over 12 weeks of follow-up, those on the experimental regimen experienced culture conversion 8 days faster on average as compared with those on the control regimen.	Quantifies treatment effect on time scale (days) Permits conclusions about faster times to culture conversion Quantifies effect over a time interval rather than at a single instance No underlying assumptions	Less familiar Requires pre-specification of time horizon of interest Requires repeated culture collections over time

**Table 2:** Primary outcomes in published RCTs

	Reference	Number of participants	Verbatim definition of stable culture conversion	Culture collection time points (weeks)	Primary outcome	Time horizons (weeks)	Reported measure(s) of treatment effect	Statistical test of treatment effect
1	Zhang, Infect Dis Poverty 2020	181	culture needed to be negative during 6,7, and 8 months of treatment, without any positive results during these 3 months	NA	proportion culture conversion	32	rate difference	Fisher’s Exact Test
2	Perumal, Clin Infect Dis 2020	197	first of 2 negative cultures at 2 different visits, without an intervening positive culture	2, 4, 6, 8	proportion culture conversion	8	rate difference	Fisher’s Exact Test
3	Lee, Lancet Infect Dis 2019	401	two consecutive negative sputum cultures. The date of culture conversion was defined as the date of the initial negative culture. Negative sputum cultures followed by contaminated cultures without subsequent positive cultures were also regarded as culture conversion.	1, 2, 3, 4, 5, 6, 7, 8, 9, 10, 11, 12, 13, 14, 15, 16	proportion culture	8	proportion difference	Fisher’s Exact Test
4	Wang, Antimicrob Agents Chemother 2018	49	NA	12, 24, 36, 48, 60, 72, 84, 96, 108, 120, 132, 144	time to culture conversion	78	difference in average time to culture conversion	Kaplan Meier Analysis
5	Aarnoutse, Antimicrob Agents Chemother 2017	150	NA	4, 6, 8, 10, 12	proportion culture conversion; time to culture conversion	4; 8	% of patients with culture conversion at 4 weeks; hazard ratio	Cox proportional hazard regression
6	Boeree, Lancet Infect Dis 2017	365	the first of two consecutive negative once-weekly sputum cultures without an intervening positive culture	1, 2, 3, 4, 5, 6, 7, 8, 9, 10, 11, 12, 14, 17, 22, 26	time to culture conversion	12	hazard ratio, median times	log rank test; PH assumption tested with Schoenfeld residuals
7	Conde, Plos One 2016	121	having two consecutive sputum specimens culture negative for M. tuberculosis, with no subsequent culture that was positive	1, 2, 3, 4, 5, 6, 7, 8, 12, 16, 20, 26	proportion culture conversion	8	proportion culture conversion	Fisher’s Exact Test
8	Tukvadze, Am J Clin Nutr 2015	192	the midpoint between the last positive Mtb sputum culture and the first negative sputum culture	2, 4, 6, 8, 12, 16	time to culture conversion	16	hazard ratio	log rank test
9	Mily, Plos One 2015	288	NA	1, 2, 3, 4, 6, 8, 10, 12, 24	proportion culture conversion	4, 8	difference in proportions, odds ratio	logistic regrsesion
10	Diacon, NEJM 2014	292	two consecutive negative liquid cultures from sputum samples that were collected at least 25 days apart and were not followed by confirmed positive cultures	1, 2, 3, 4, 5, 6, 7, 8, 10, 12, 14, 16, 18, 20, 22, 24	time to culture conversion	24	median times, hazard ratio, proportion culture conversion	log tank test
11	Carroll, Antimicrob Agents Chemother 2013	35	conversion date was defined as the first date of three consecutive negative tests at least 1 month apart	NA	time to culture conversion	8	median times	log rank test
12	Lee, N Engl J Med 2012	41	negative sputum samples on solid medium for 3 consecutive weeks	1, 2, 3, 4, 5, 6, 7, 8, 9, 10, 11, 12, 13, 14, 15, 16	time to culture conversion	16	cumulative proportions achieving culture conversion (using KM estimator)	Wilcoxon test
13	Dorman, J Infect Dis 2012	531	first of 2 consecutive culturenegative sputum sample collected on nonconsecutive days that were not followed by a culture-positive specimen	2, 4, 6, 8	proportion culture conversion; time to culture conversion	8; 8	difference in proportions	Pearson Chi Square test with Yates correction; log rank test
14	Gler, N Engl J Med 2012	481	first of five or more consecutive weekly cultures that were negative for growth of M. tuberculosis without subsequent positive cultures	1, 2, 3, 4, 5, 6, 7, 8, 9, 10, 11, 12	proportion culture conversion	8	proportion culture conversion	Cochran-Mantel-Haenszel test

**Table 3:** Primary outcomes in registered RCTs

Reference	Number of participants	Verbatim definition of stable culture conversion	Culture collection time points (weeks)	Primary Outcome	Time horizons (weeks)	Repoiicd measure(s) of treatment effect	Statistical test of treatment effect
NCT04311502, November 2020	185	the first of two (consecutive or non-consecutive) negative sputum cultures without an intervening positive culture	NA	time to culture conversion	12	NA	NA
NCT04575519, November 2020	354	at least two consecutive negative cultures for M. tuberculosis at least 4 weeks apart during the first 24 weeks of TB treatment	NA	time to culture conversion	24	NA	NA
NCT04504851, August 2020	154	first of two consecutive negative sputum cultures	NA	time to culture conversion	8	NA	NA
NCT03702738, March 2019	110	NA	NA	time to culture conversion	26	median times	NA
NCT03338621, July 2018	455	NA	NA	time to culture conversion	8	NA	NA
NCT02589782, January 2017	630	NA	NA	proportion culture conversion	8	NA	NA
NCT03281226, December 2016	50	NA	NA	proportion with biological intolerability or AE	26	NA	NA
NCT02619994, January 2016	238	NA	NA	proportion treatment success	104	NA	NA
NCT02256696, April 2015	183	NA	NA	time to culture conversion	12	NA	NA
NCT02454205, November 2015	154	NA	NA	proportion treatment success	104	NA	NA
NCT01918397, January 2015	111	first of two successive negative cultures one study visit apart that are not followed by a culture-positive specimen with 28 weeks of treatment initiation	2, 4, 6, 8, 10, 12, 16, 20, 24	time to culture conversion	28	NA	NA

## Box

Culture conversion endpoints are common in phase IIb TB treatment trials, but there is variability in definition, analysis, and interpretation. Harmonization of these attributes is important for progress in TB therapeutics. Restricted mean survival times may complement other analytical methods.
