# Supplementary material for: Treatment effect measures for culture conversion endpoints in phase IIb tuberculosis treatment trials
Source: Clin Infect Dis. Author manuscript; Available in PMC 2021 Dec 13. (PMC8664460; doi:10.1093/cid/ciab576)
Supplement: Supplementary Material [file EMS128113-supplement-Supplementary_Material.pdf]

Click here to access/download;Supplemental data/Appendix-published online only;CID-107795_appendix.pdf

**Appendix Text 1: Additional information on RMST**

The restricted mean survival times (RMST) is given by the area under the survival curve, $S(t)$, from randomization to time horizon $\tau$. The Kaplan-Meier estimator, $\hat{S}(t)$,is used to estimate the RMST. Thus $\widehat{RMST} = \hat{\mu}_\tau = \int_0^\tau \hat{S}(t)\mathrm{du}$ with variance derived from Greenwood's formula:

$$\mathrm{Var}(\hat{\mu}_\tau) = \hat{\sigma}^2(\hat{\mu}_\tau) = \sum_{i=1}^{D}\left[\int_0^\tau \hat{S}(t)\mathrm{dt}\right]^2 \frac{d_i}{Y_i(Y_i - d_i)}.$$

The difference in RMST, denoted $\delta_\tau$, is a natural treatment effect measure to compare the times to culture conversion between two arms. The point estimate and associated (1-α)% confidence interval are given by

$$\hat{\delta}_\tau = \int_0^\tau \hat{S}_E(t)\mathrm{dt} - \int_0^\tau \hat{S}_C(t)\mathrm{dt}$$

and

$$\hat{\delta}_\tau \pm z_{1-\frac{\alpha}{2}}\sqrt{\hat{\sigma}^2\left(\hat{\mu}_{E,\tau}\right) + \hat{\sigma}^2(\hat{\mu}_{C,\tau})}, \text{ respectively.}$$

The time horizon for restriction should be pre-specified and driven by clinical relevance. However, the RMST is mathematically estimable at any time horizon before the last observed follow-up time; either an event or censoring time. Therefore, to calculate the difference in RMST for two randomization groups, the time horizon should be less than or equal to the minimum of the last follow-up times across the two groups.

**Appendix Text 2: Note about data reconstruction**

Without access to the raw trial data, we relied on reconstruction methods for our reanalysis. We therefore followed the Kaplan-Meier curves and axes exactly as printed and published in the final manuscript of Boeree, LID 2017. While reconstruction of data has repeatedly demonstrated accuracy, it is not a perfect tool.

The reconstruction process involves tracing the x and y-axes and the Kaplan-Meier curves for each arm of interest using the figure printed in the published manuscript file. These figures are often not perfectly accurate and may lead to some discrepancies. In the case of the MAMS trial presented in the main text, we have reported the cumulative probability of culture conversion estimate for the control arm at 11.82 weeks instead of 12.00 weeks in order to match the reported values from the published manuscript more closely. The authors believe this discrepancy is due to misalignment between the printed x-axis and the Kaplan-Meier curve. It is important to note that the estimated difference in RMST remains the same (8 days) when estimated for the time horizons of 11.82 and 12.00 weeks.

**Appendix Text 3: Systematic selection of studies**

We identified trials published between January 2011 and October 2020 and trials registered with a study start date between January 2011 and January 2021. One author (IRW) reviewed each abstract and full text to determine eligibility for published manuscript inclusion. We required that the manuscript reported a primary analysis of a phase II RCT, evaluated a comparison of treatments for pulmonary TB and, used a culture conversion primary outcome. From each eligible manuscript, we extracted the primary outcome details and noted any culture conversion secondary outcomes. We extracted the pre-specified analysis method, time horizon of interest, and definition of stable culture conversion, if available. We recorded trial characteristics and extracted culture collection time points. Protocols and statistical analysis plans were searched for additional information, if available. For trial registrations, we required that the planned trial be 'actively recruiting', 'not yet recruiting', 'active but not recruiting', or 'enrolling by invitation', have a phase II design and assess treatment for pulmonary TB, and be designed for a culture conversion primary outcome. We excluded pharmacokinetic, early bactericidal activity (EBA), and dose finding studies. For eligible registrations, we extracted the anticipated number of participants, definition of stable culture conversion, and culture collection time points. We noted the primary and secondary outcomes related to culture conversion including the time horizons of interest and the planned analysis method, if provided.

**Appendix Text 4: Pub Med search equation**

(2011/01/01:3000/12/31[Date - Publication] AND "Tuberculosis"[Title/Abstract] AND "culture"[Title/Abstract] AND ("randomized"[Title/Abstract] OR "randomly"[Title/Abstract]) AND ("treatment"[Title/Abstract] OR "regimen"[Title/Abstract])) NOT "protocol"[Title] NOT ("meta-analysis"[Title/Abstract] OR "meta analysis"[Title/Abstract] OR "systematic review"[Title/Abstract])

## Appendix Table 1: Secondary outcomes in published RCTs

| Reference | Number of participants | Verbatim definition of stable culture conversion | Culture collection time points (weeks) | Secondary outcome | Time horizons (weeks) | Reported measure(s) of treatment effect | Statistical test of treatment effect |
|---|---|---|---|---|---|---|---|
| Zhang, Infect Dis Poverty 2020 | 181 | culture needed to be negative during 6,7, and 8 months of treatment, without any positive results during these 3 months | NA | - | - | - | - |
| Perumal, Clin Infect Dis 2020 | 197 | first of 2 negative cultures at 2 different visits, without an intervening positive culture | 2, 4, 6, 8 | time to culture conversion | 8 | hazard ratio, median times | Gehan-Breslow-Wilcoxon test |
| Lee, Lancet Infect Dis 2019 | 401 | two consecutive negative sputum cultures. The date of culture conversion was defined as the date of the initial negative culture. Negative sputum cultures followed by contaminated cultures without subsequent positive cultures were also regarded as culture conversion. | 1, 2, 3, 4, 5, 6, 7, 8, 9, 10, 11, 12, 13, 14, 15, 16 | time to culture conversion | 24 | median times | log rank test |
| Wang, Antimicrob Agents Chemother 2018 | 49 | NA | 12, 24, 36, 48, 60, 72, 84, 96, 108, 120, 132, 144 | - | - | - | - |
| Aarnoutse, Antimicrob Agents Chemother 2017 | 150 | NA | 4, 6, 8, 10, 12 | - | - | - | - |
| Boeree, Lancet Infect Dis 2017 | 365 | the first of two consecutive negative once-weekly sputum cultures without an intervening positive culture | 1, 2, 3, 4, 5, 6, 7, 8, 9, 10, 11, 12, 14, 17, 22, 26 | proportion culture conversion | 12 | cumulative proportions achieving culture conversion (using KM estimator) | not tested |
| Conde, Plos One 2016 | 121 | having two consecutive sputum specimens culture negative for M. tuberculosis, with no subsequent culture that was positive | 1, 2, 3, 4, 5, 6, 7, 8, 12, 16, 20, 26 | time to culture conversion | 26 | difference in median times | log rank test; Wilcoxon two sample rank sum test |
| Tukvadze, Am J Clin Nutr 2015 | 192 | the midpoint between the last positive Mtb sputum culture and the first negative sputum culture | 2, 4, 6, 8, 12, 16 | proportion culture conversion | 8 | cumulative proportions achieving culture conversion (using KM estimator) | |
| Mily, Plos One 2015 | 288 | NA | 1, 2, 3, 4, 6, 8, 10, 12, 24 | time to culture conversion | 24 | none reported | log rank test |
| Diacon, NEJM 2014 | 292 | two consecutive negative liquid cultures from sputum samples that were collected at least 25 days apart and were not followed by confirmed positive cultures | 1, 2, 3, 4, 5, 6, 7, 8, 10, 12, 14, 16, 18, 20, 22, 24 | - | - | - | - |
| Carroll, Antimicrob Agents Chemother 2013 | 35 | conversion date was defined as the first date of three consecutive negative tests at least 1 month apart | NA | - | - | - | - |
| Lee, N Engl J Med 2012 | 41 | negative sputum samples on solid medium for 3 consecutive weeks | 1, 2, 3, 4, 5, 6, 7, 8, 9, 10, 11, 12, 13, 14, 15, 16 | - | - | - | - |
| Dorman, J Infect Dis 2012 | 531 | first of 2 consecutive culture-negative sputum sample collected on nonconsecutive days that were not followed by a culture-positive specimen | 2, 4, 6, 8 | - | - | - | - |
| Gler, N Engl J Med 2012 | 481 | first of five or more consecutive weekly cultures that were negative for growth of M. tuberculosis without subsequent positive cultures | 1, 2, 3, 4, 5, 6, 7, 8, 9, 10, 11, 12 | time to culture conversion | 5 | hazard ratio | not tested |

## Appendix Table 2: Secondary outcomes in registered RCTs

| Reference | Number of participants | Verbatim definition of stable culture conversion | Culture collection time points (weeks) | Secondary Outcome | Time horizons (weeks) | Reported measure(s) of treatment effect | Statistical test of treatment effect |
|---|---|---|---|---|---|---|---|
| NCT04311502, November 2020 | 185 | the first of two (consecutive or non-consecutive) ı | NA | proportion culture conversion | 8, 12 | NA | NA |
| NCT04575519, November 2020 | 354 | at least two consecutive negative cultures for M. t | NA | time to culture conversion | 8, 16 | NA | NA |
| NCT04504851, August 2020 | 154 | first of two consecutive negative sputum cultures | NA | time to culture conversion; proportion culture conversion | 12; 8 | NA | NA |
| NCT03702738, March 2019 | 110 | NA | NA | - | - | - | - |
| NCT03338621, July 2018 | 455 | NA | NA | proportion culture conversion | 4, 6, 12, 17 | NA | NA |
| NCT02589782, January 2017 | 630 | NA | NA | time to culture conversion | 108 | median times | NA |
| NCT03281226, December 2016 | 50 | NA | NA | proportion culture conversion | 8 | NA | NA |
| NCT02619994, January 2016 | 238 | NA | NA | time to culture conversion; proportion culture conversion | 104; (8, 26) | median times | log rank test |
| NCT02256696, April 2015 | 183 | NA | NA | proportion culture conversion | 8, 12 | NA | NA |
| NCT02454205, November 2015 | 154 | NA | NA | proportion culture conversion | 26-76 | NA | NA |
| NCT01918397, January 2015 | 111 | first of two successive negative cultures one study visit apart that are not followed by a culture-positive specimen with 28 weeks of treatment initiation | 2, 4, 6, 8, 10, 12, 16, 20, 24 | - | - | - | - |
